# Hibernation is associated with increased survival and the evolution of slow life histories among mammals

**DOI:** 10.1098/rspb.2011.0190

**Published:** 2011-03-30

**Authors:** Christopher Turbill, Claudia Bieber, Thomas Ruf

**Affiliations:** Department of Integrative Biology and Evolution, Research Institute of Wildlife Ecology, University of Veterinary Medicine Vienna, Savoyenstrasse 1, 1160 Vienna, Austria

**Keywords:** hibernation, life history, life span, longevity, mammal

## Abstract

Survival probability is predicted to underlie the evolution of life histories along a slow–fast continuum. Hibernation allows a diverse range of small mammals to exhibit seasonal dormancy, which might increase survival and consequently be associated with relatively slow life histories. We used phylogenetically informed GLS models to test for an effect of hibernation on seasonal and annual survival, and on key attributes of life histories among mammals. Monthly survival was in most cases higher during hibernation compared with the active season, probably because inactivity minimizes predation. Hibernators also have approximately 15 per cent higher annual survival than similar sized non-hibernating species. As predicted, we found an effect of hibernation on the relationships between life history attributes and body mass: small hibernating mammals generally have longer maximum life spans (50% greater for a 50 g species), reproduce at slower rates, mature at older ages and have longer generation times compared with similar-sized non-hibernators. In accordance with evolutionary theories, however, hibernating species do not have longer life spans than non-hibernators with similar survival rates, nor do they have lower reproductive rates than non-hibernators with similar maximum life spans. Thus, our combined results suggest that (i) hibernation is associated with high rates of overwinter and annual survival, and (ii) an increase in survival in hibernating species is linked with the coevolution of traits indicative of relatively slow life histories.

## 1. Introduction

The existence of a trade-off between lifespan and reproduction is central to the concept of an evolved life history strategy [1–3]. With limited resources, an organism cannot simultaneously maximize both of these traits but must balance investment in survival versus offspring to maximize its lifetime reproductive fitness. Moreover, in stable populations, survival and birth rates must be inversely related [4]. The evolution of life history strategies therefore is constrained along a slow–fast continuum, in which species with slow life histories generally have higher survival rates, live longer maximum life spans, mature at older ages and produce fewer young per year compared with species with fast life histories [5–9].

Hibernation is a distinctive trait that could affect survival; hence the evolution of mammalian life histories. All three mammalian subclasses and around half of all orders contain hibernating species, although most are relatively small (median body weight: 85 g) [10]. Hibernation is viewed as an energy-saving adaptation that allows small endotherms to reside year-round in highly seasonal climates. There appear to be conflicting views, however, regarding the mortality risk over the winter hibernation season [11,12]. Yet, even very small hibernators can store enough fat and have low enough metabolic rates to remain dormant for up to an entire year [13,14]. By allowing long periods of inactivity, usually while hidden in underground burrows or caves, hibernation could also largely reduce the risk of predation. This is a plausible explanation, for example, for the prolonged summer dormancy recently documented in edible dormice (*Glis glis*). In this case, hibernation clearly is unrelated to energetic constraints, but probably functions to increase survival by eliminating predation from owls during years when most individuals skip reproduction [15]. Indeed, a growing number of quantitative studies have documented remarkably high overwinter survival rates in hibernating species [11,16–18]. Hibernation is a significant factor in explaining variation in maximum recorded lifespan among bat species [19], yet surprisingly there has been no investigation of whether it has a general effect on annual survival and the evolution of life histories among all mammals.

We first reviewed the published literature on seasonal variation in survival within populations of hibernating mammals. This showed that the hibernation season is associated with very high rates of monthly survival. We then fitted phylogenetically informed regression models to test whether the trait of hibernation has an effect on annual survival, maximum lifespan, annual reproductive output, age at maturity and generation time among a large sample of mammal species. Our comparative analyses reveal hibernation is associated with an increase in annual survival relative to body mass. Moreover, as predicted by evolutionary theories, higher survival rates appear to be linked to the evolution of a slow pace of life histories in hibernating mammals.

## 2. Material and methods

### (a) Seasonal survival

We found 22 published studies providing estimates of survival probability over the hibernation and active season or at a finer resolution over the entire year for populations of hibernating mammal species. In addition, we included new data on seasonal survival probability estimated for two populations of the edible dormouse, *G. glis*. In total, we collated data on seasonal variation in survival probability for 40 groups of individuals (i.e. males, females, juveniles, adults) from 19 species of hibernating mammals (see the electronic supplementary material, table S1).

### (b) Annual survival and other life history variables

We collated a dataset of annual survival probability by combining published datasets [20–22], supplemented with data from those studies we cited in our analysis of seasonal survival. For each species, we derived a single median value of estimated annual survival probability.

We also obtained values for annual reproductive rate (litter size multiplied by litters per year) and age at sexual maturity from a published dataset for mammal species [23]. We calculated values for generation time (*T*_g_), using the function: *T*_g_ = age of maturity + (survival/(1 − survival)) [24]. For maximum life span, we combined data from the PanTHERIA [23] and AnAge [25] datasets, with values from AnAge taken in cases of discrepancy because in general these values appeared to be more accurate. We further supplemented these data with values from several other sources [5,19,26–30]. Data for the reproductive rate of two species of hibernating hamsters (*Cricetus cricetus* and *Mesocricetus auratus*) were excluded from our analysis because these values are from laboratory colonies and are unlikely to be representative of natural populations. We also removed data for age at sexual maturity for the primates because they differed radically as a group from other mammals.

We classified each species in our analyses either as a hibernator, if they were known to hibernate in at least part of their geographical range, or a non-hibernator, if they were not known to hibernate [10,19,28,31–35] (see the electronic supplementary material, table S2). We defined hibernation as a period of seasonal dormancy accompanied by multi-day periods of hypometabolism. We excluded data for potential hibernating (i.e. ‘denning’ bears) species from the order Carnivora (nevertheless, very similar results were obtained when we included them as hibernators) and five species from other orders because of uncertainty regarding their classification as hibernators.

### (c) Statistical analysis

All analyses were carried out using R v. 2.12.1 [36]. Survival rates (in the interval 0–1) were arcsine square root transformed to obtain normally distributed data. Differences in survival between the seasons were initially analysed using a Wilcoxon signed-rank test on subgroups (e.g. adults) of all 40 samples. We then tested for effects of season on survival (species means), and hibernation on adult annual survival, maximum life span, reproductive rate, age at sexual maturity and generation time by fitting phylogenetically informed generalized least squares (PGLS) models. Model selection (see below) was based on minimizing the value of Akaike's information criterion (AIC). We present the results of PGLS models as regression tables. Coefficients in these tables are the common intercept, intercept shifts associated with the levels of factorial predictors (i.e. ‘hibernation’), regression slopes for continuous predictors and differences in slopes for interactions. We also give corresponding *t*-values and *p*-values for each coefficient.

We fitted PGLS models using function ‘gls’ in R. In these models, phylogenetic correlation between taxonomically related species is used for sample weighting because data-points of closely related species are not entirely independent. We used an updated version [37] of the mammalian supertree [38] to set up correlation structures. For each dataset analysed, tips for unavailable species were dropped from this tree. Please note that while the species names used as tip labels in these trees follow the partially outdated nomenclature given in Wilson & Reader [39], the actual phylogenetic relationships are based on up-to-date analyses [37,38]. To compute phylogenetic correlation structures, we used the correlation classes implanted in the R-library ‘ape’ [40]. Initial trials (using both dated and equal branch length trees) showed that for all response variables investigated, using the covariance matrix ‘corPagel’ [41,42] led to much lower estimates of model AIC than any other correlation class implemented in package ape (i.e. Brownian models [43], the Ornstein–Uhlenbeck process [44], Grafen's method [45] and the ‘ACDC’ (accelerated/decelerated) model [46]). All phylogenetically informed models also led to much smaller AIC values than ordinary least squares (OLS) analysis. Pagel's *λ* accounts for the phylogenetic covariance between response and explanatory variables. This method avoids the errors associated with assuming complete phylogenetic independence (*λ* = 0, equivalent to OLS analyses) or the overcorrecting caused by assuming complete phylogenetic covariance (*λ* = 1, equivalent to phylogenetically independent contrasts) [47]. Maximum-likelihood estimates of Pagel's *λ* showed a strong phylogenetic signal in all variables, while randomizing tip labels of trees yielded values of *λ* that varied around 0 in all cases. Therefore, we do not present alternative models, such as OLS.

To obtain approximately linear relations, maximum life span, body mass and reproductive rates were log-transformed. To investigate the relation of maximum life span to reproductive rate, we used the square root of maximum life span, which yielded a better linear relation than log maximum life span. Log body mass was included as a covariate in all models. For several traits, visual inspection of the data indicated that means of variables in bats clearly differed from those of all other mammals. Therefore, we used an additional factor called ‘bat’ in all models tested initially (but this factor was sometimes removed during model selection). Allowing for this offset in Chiroptera also means that any detected effects of hibernation on traits were not merely caused by deviations of hibernating bats from other mammals. Hibernation is largely (although not solely) restricted to higher latitudes, so in all full models we included a value of latitude for each species that is midway between the southern and northern extent of its geographical range [23]. However, this latitude variable was not retained in any of the final best models.

## 3. Results

### (a) Seasonal variation in survival

**Figure 1.**
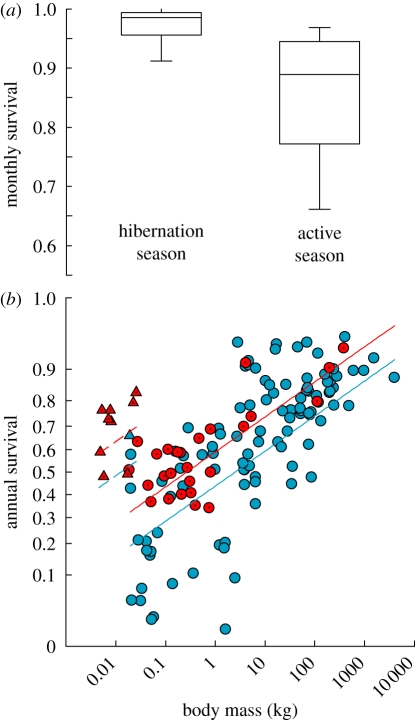
(*a*) Monthly survival probability of adults estimated over the hibernation and active season for 19 hibernating mammal species (box plots show the median (*line*), 25% and 75% (*box*), and 10% and 90% (*whiskers*) percentiles), and (*b*) estimates of annual survival probability of adult mammals as a function of body mass and the PGLS model-predicted regression lines (see table 1 for regression results). Hibernation had a positive effect on annual survival probability (*t* = 2.12; *p* = 0.036). Filled blue circles and solid blue line, non-hibernators; filled blue triangles and dashed blue line, non-hibernators (bats); filled red circles solid line, hibernators; filled red triangles and dashed red line, hibernators (bats).

Monthly survival probability of adults (*n* = 32) was significantly higher and much less variable during the hibernation season (0.970 ± 0.033 s.d.) (i.e. 97.0% probability of survival over each month) than during the active season (0.845 ± 0.136; Wilcoxon signed-rank test, *p* < 0.001; figure 1*a*). Overall, monthly survival probability was higher (by a median of 0.08) during the hibernation season in 37 (93%) out of the 40 within-group (i.e. sex and age) comparisons. When survival estimates for sexes and ages were averaged for each of the 19 species, a phylogenetically informed model included a significant and strong effect of season on survival (*t* = 41.6, *p* < 0.001). For juveniles, which represent only a subset of the data (*n* = 8), monthly survival was also higher during hibernation (0.948 ± 0.031) than during the active season (0.884 ± 0.077; *p* = 0.05).

### (b) Annual survival

Our best PGLS model of variation in adult annual survival probability among mammal species indicated a strong relationship with body mass, as well as additive positive effects of both bats and hibernation (*t* = 2.12; *p* = 0.036; table 1 and figure 1*b*). The PGLS model coefficients suggested that hibernators on average have approximately 15 per cent higher annual survival compared with non-hibernators of equivalent body mass.

**Table 1.** Regression results for the best PGLS model explaining variation in arcsine square root annual survival probability among mammal species (*n* = 141, Pagel's *λ* = 0.79).

fixed effect	coefficient	s.e.	*t*-value	*p*-value
intercept	0.239	0.151	1.59	0.1150
bat	0.359	0.166	2.16	0.0324
hibernation	0.182	0.086	2.12	0.0363
log_10_ mass (g)	0.164	0.024	6.80	<0.0001

### (c) Maximum life span

**Figure 2.**
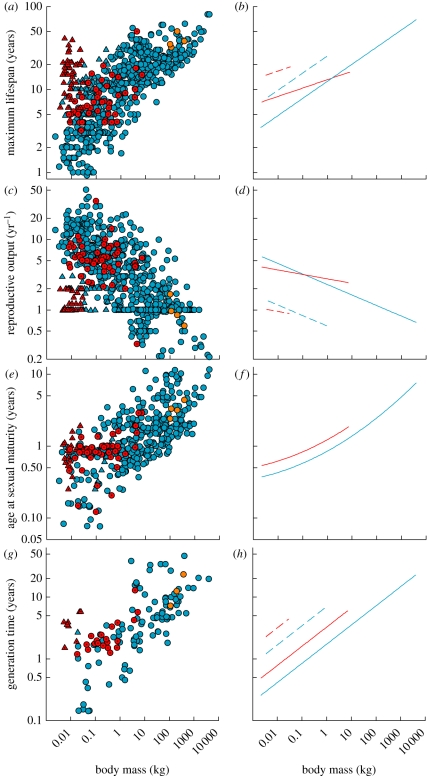
(*a*,*b*) Maximum life span, (*c*,*d*) annual reproductive output, (*e*,*f*) age at sexual maturity and (*g*,*h*) generation time of mammal species as a function of their body mass. Hibernation affected the relationship of each life-history attribute with body mass (see table 2 for regression results). Shown are the raw data and the PGLS model-predicted regression lines. Filled blue circles solid lines, non-hibernators; filled blue triangles dashed lines, non-hibernators (bats); filled red circles solid lines, hibernators; filled red triangles dashed lines, hibernators (bats); filled orange circles, bears.

We found a significant effect of hibernation on the slope of the relationship between maximum life span and body mass (*t* = −3.02, *p* = 0.003) and also a positive additive effect of bats (table 2 and figure 2*a*,*b*). The PGLS model suggested the slope of the relationship with body mass is much flatter for hibernators than for non-hibernators, with the model-predicted regression lines for non-bats intersecting at approximately 1.5 kg. Below this body mass, hibernation has an increasingly positive effect on maximum life span.

**Table 2.** Regression results for the best phylogenetically informed generalized least-squares models explaining variation in maximum life span, annual reproductive rate, age at sexual maturity and generation time (see §2 for definition) among mammal species.

fixed effect	coefficient	s.e.	*t*-value	*p*-value
log_10_ maximum life span (years) (*n* = 652, Pagel's *λ* = 0.76)				
intercept	0.480	0.127	3.77	0.0020
bat	0.306	0.112	2.72	0.0066
hibernation	0.335	0.070	4.81	<0.0001
log_10_ mass, (g)	0.206	0.013	16.23	<0.0001
hibernation × log_10_ mass, (g)	−0.105	0.035	−3.02	0.0026
log_10_ annual reproductive output (*n* = 649, *λ* = 0.94)				
intercept	0.807	0.200	4.05	0.0001
bat	−0.589	0.175	−3.36	0.0008
hibernation	−0.177	0.075	−2.36	0.0190
log_10_ mass, (g)	−0.149	0.015	−9.67	<0.0001
hibernation × log_10_ mass, (g)	0.090	0.039	2.20	0.0279
log_10_ age at sexual maturity (*n* = 543, *λ* = 0.93)				
intercept	2.11	0.209	10.10	<0.0001
hibernation	0.159	0.052	3.07	0.0023
log_10_ mass, (g)	0.067	0.051	1.30	0.1938
log_10_ mass^2^, (g)	0.021	0.008	2.72	0.0067
log_10_ generation time (*n* = 128, *λ* = 0.77)				
intercept	−0.696	0.221	−3.15	0.0021
bat	0.610	0.243	2.51	0.0135
hibernation	0.278	0.128	2.17	0.0320
log_10_ mass, (g)	0.311	0.036	8.56	<0.0001

### (d) Annual reproductive output

We found a significant effect of hibernation on the slope of the relationship between log annual reproductive rate and body mass (*t* = 2.20, *p* = 0.028) and a negative effect of bats (table 2 and figure 2*c*,*d*). The PGLS model-predicted regression lines for hibernating and non-hibernating species intersected at a body mass of 0.115 kg, which is 30 g above the median weight of hibernating mammal species [10]. Below this body mass, the model predicts an increasingly negative effect of hibernation on annual reproductive output. The model also suggests a positive effect of hibernation on annual reproductive output in the largest hibernators; however, this should be given a low weighting because of the few data in this range of body masses. It should also be noted that the regression of non-hibernators against body mass using a phylogenetically informed covariance structure differs somewhat from the relationship suggested by the raw data. Among rodents weighing less than 100 g, for example, the average annual reproductive output of hibernating species (7.9 ± 1.7, *n* = 18) is approximately half that of non-hibernators (14.3 ± 1.2, *n* = 60).

### (e) Age at sexual maturity and generation time

Age at sexual maturity increased with body mass, and hibernation had a significant positive effect on the elevation of this relationship (*t* = 3.07, *p* = 0.002; table 2 and figure 2*e*,*f*). Similarly, when adjusted for body mass, generation time was higher in bats and additionally elevated in hibernators (*t* = 2.17, *p* = 0.032; table 2 and figure 2*g*,*h*).

### (f) Interrelations among survival, maximum life span and reproductive rate

**Figure 3.**
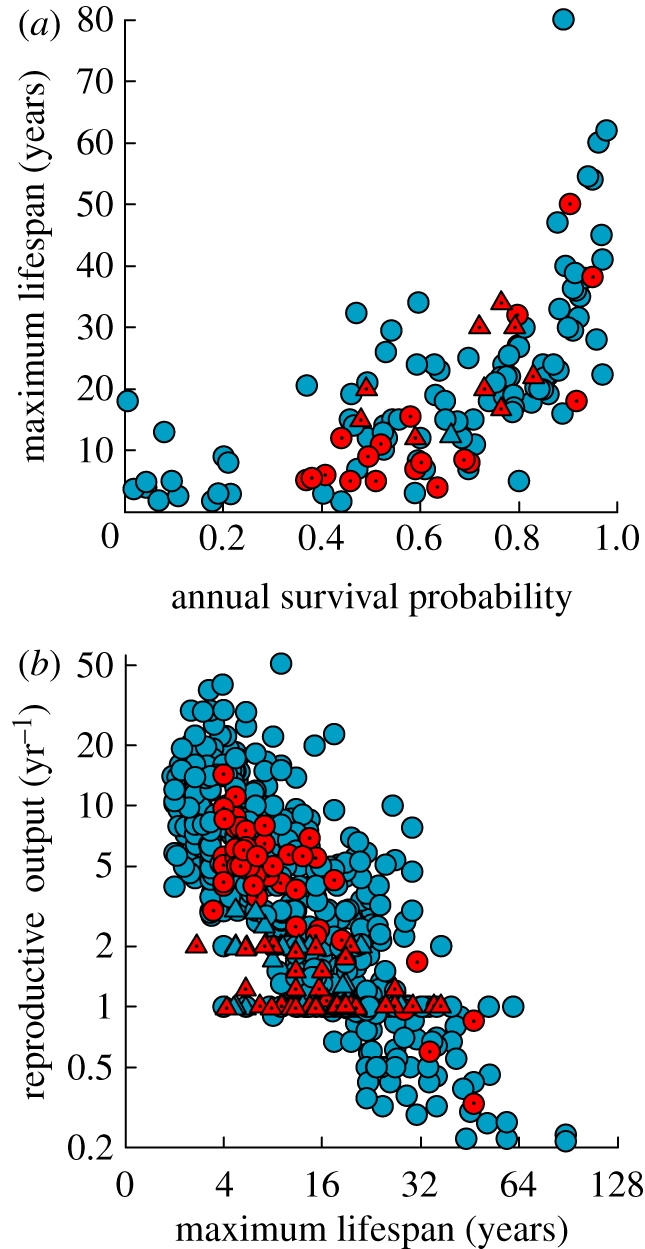
Hibernation did not affect (*p* = 0.35) the relationships between (*a*) maximum lifespan and annual survival probability, and (*b*) annual reproductive output and maximum lifespan (*t* = 0.43, *p* = 0.67) among mammal species. Filled blue circles, non-hibernators; filled blue triangles, non-hibernators (bats); filled red circles, hibernators; filled red triangles, hibernators (bats).

Hibernation did not influence (*p* = 0.35) the positive relationship between maximum life span and annual survival probability (*t* = 2.65, *p* = 0.009; figure 3*a*), nor (*p* = 0.67) that between annual reproductive output and maximum life span among mammal species (*t* = 12.4, *p* < 0.001; figure 3*b*). In other words, whereas hibernation was an important factor explaining variation in life history traits when they were expressed relative to body mass, its effect was not apparent when these traits were expressed relative to survival or among themselves.

## 4. Discussion

### (a) Survival

Our review shows the hibernation season is associated with a high probability of survival [11,12,16,17]. Indeed, a small mammal is five times more likely to die over each month of the active season compared with during hibernation. Survival during hibernation is probably even higher than reported values because some mortalities occurring in the active season tend to be attributed to the hibernation season [17,48]. Even though studies on seasonal survival of hibernators are biased towards rodents, the data available for three other mammalian orders suggest our conclusions could be generalized to other hibernating mammals.

Predation is a major cause of mortality in small mammals and the risk of predation is related to levels of activity [49–52]. Hibernating mammals are inactive for up to nine months of the year, during which they typically hide in a sealed burrow or other protected shelter. For weeks at a time, they remain motionless, have a cold body temperature and emit few metabolic odours. Inactivity combined with a lowered metabolism appears to be an effective way to avoid predation [53]. Hibernators completely evade predation by birds, which are a major threat for small mammals. Reports of great tits (*Parus major*) predating hibernating bats are probably exceptional [54]. The risk posed by mammalian predators is also much reduced [17,55], although badgers are known to excavate hibernating ground squirrels [56,57].

Most hibernating species are isolated from external weather conditions, which can reduce survival over-winter in non-hibernating species [58–61]. For juveniles, pre-hibernation body mass is a strong predictor of overwinter survival [62–64]. Late-born juveniles, especially, have trouble gaining sufficient energy for both growth and deposition of pre-hibernation fat stores, and consequently they can suffer relatively high overwinter mortalities [64,65].

Hibernation allows small endotherms to overcome the severe energetic challenge imposed by winter in seasonal climates. However, individuals sometimes hibernate even when food energy is available (e.g. [15]) and, in the same environments, other small mammals can be active throughout the winter. We suggest that a past focus on the remarkable metabolic and thermal physiology of hibernation has partly obscured its broader ecological significance. Hibernation is a prerequisite for small mammals to employ seasonal dormancy, which greatly increases the probability of surviving while environmental conditions are sub-optimal for reproduction. An ability to forego activity for up to nine months of the year, even when food is available, could in itself be an important purpose of hibernation. In other words, we suggest that predator avoidance, rather than energy savings, may have been the primary selective force for the evolution of hibernation.

We found that hibernating mammals generally also have higher *annual* survival than predicted for their body mass. This effect is robust because we could detect it despite the inherent variability in estimates of survival for wild populations. We expected hibernation could particularly benefit the survival of the smallest species, which probably are more vulnerable to predation and starvation during winter than larger species, but we did not find a significant effect of hibernation on the slope of the relationship between survival and body mass. Nevertheless, there is an apparent trend in the data for hibernation to have a greater positive effect on survival in smaller species.

### (b) Traits indicative of the pace of life histories

Hibernation has a significant influence on each of the key life history traits included in our analyses and these effects become more pronounced in smaller species. Mammals capable of hibernation generally have longer maximum recorded lifespans than predicted for their body mass. Our model predicts a 50 g hibernator, for example, has a potential maximum life span that is approximately 50 per cent or 2.8 years greater than its non-hibernating counterpart. A positive effect of hibernation on maximum life span was shown previously among bat species [19]. Our results suggest this conclusion is generally applicable to all hibernating mammals. We interpret maximum recorded life span as an approximate index of the rate of senescence in survival, with which it is strongly correlated among vertebrate animals [9,66]. As an index of senescence, maximum life span is not necessarily linked to estimates of survival in wild populations, which chiefly are measured in young adults and reflect environmental causes of mortality [67]. Estimates of maximum life span increase with sample size, but this effect diminishes rapidly in populations with even low rates of senescence [68,69]. Critically, sample sizes are not likely to be systematically greater in hibernating species. Our results therefore suggest that, for their size, hibernating mammals have relatively slow rates of senescence in survival. When plotted as a function of annual survival, however, the maximum life span of hibernating species becomes indistinguishable from non-hibernators. That is, hibernators have a maximum life span matching the age expected from their relatively high rates of survival.

Our analyses also suggested an effect of hibernation on other key life history traits. Small hibernators generally have lower reproductive rates than predicted for their body mass. Whereas, when annual reproductive output is plotted against maximum life span, hibernating species are indistinguishable from other mammals. We also found a positive effect of hibernation on age of sexual maturity, which is strongly correlated with the pace of life histories [9,70–73]. Small non-hibernating mammals generally reach sexual maturity after several months of age, yet many hibernators delay maturity until after the following spring at approximately 1 year of age. Finally, we also found that hibernation has a positive effect on generation time, which can be calculated from age of maturity and estimated mean life span. Generation time has been shown to be a strong predictor of other life history traits [24], including the age at onset of senescence [71].

Our combined analyses suggest a link between the positive effect of hibernation on survival and the evolution of a relatively slow life history in hibernating mammals. This interpretation is in agreement with evolutionary theories of ageing, which predict survival rates of adult individuals to determine the optimal level of an apparent trade-off between somatic maintenance (i.e. senescence) and reproduction [3,67,73,74]. In support of this theory, survival rates of bird and mammal populations are strongly correlated with variation in the rate of senescence in survival [66,67] and the pace of other life-history variables [7,8,71,73]. Other traits assumed to reduce the risk of predation, such as flight, arboreality, eusociality or chemical defences, have also been associated with a relatively long maximum life span [75–79]. Unlike previous studies, however, our analyses show a pervasive effect of hibernation on survival, rate of reproduction, age of maturity and generation time, which are indicative of the coevolution of traits towards a slower life history strategy.

Our study suggests a link in the direction of hibernation → survival → life history, but we cannot be certain of the direction of cause and effect among these traits. Hibernation clearly is an energetic requirement for many small mammals, such as insectivorous bats, to reside year-round at temperate latitudes. The need to fatten prior to hibernation, for example, could restrict investment in reproduction and hence lead to demographic changes that drive the evolution of correlated traits such as survival and rate of senescence. Nevertheless, many resident passerine birds and small mammals do not hibernate over the temperate winter season. Hibernators also can remain dormant well into the season when food is available [15], and hibernating species are not limited to cold temperate climates [80]. This supports a general view of hibernation as a physiological mechanism permitting small mammals to remain dormant and increase survival when conditions are not optimal for reproduction. Our comparative analyses suggest that, regardless of the direction of causal effects, an increase in survival in hibernating mammals appears to have coevolved with a relatively slow life history.
